# A new method to attribute differences in total deaths between groups to population size, age structure and age-specific mortality rate

**DOI:** 10.1371/journal.pone.0216613

**Published:** 2019-05-10

**Authors:** Xunjie Cheng, Liheng Tan, Yuyan Gao, Yang Yang, David C. Schwebel, Guoqing Hu

**Affiliations:** 1 Department of Epidemiology and Health Statistics, Xiangya School of Public Health, Central South University, Changsha, China; 2 Department of Biostatistics, College of Public Health and Health Professions, and Emerging Pathogens Institute, University of Florida, Gainesville, Florida, United States of America; 3 Department of Psychology, University of Alabama at Birmingham, Birmingham, AL, United States of America; Bielefeld University, GERMANY

## Abstract

**Background:**

Two decomposition methods have been widely used to attribute death differences between two populations to population size, age structure of the population, and age-specific mortality rate (ASMR), but their properties remain uninvestigated.

**Methods:**

We assess how the two established decomposition methods yield varying results with three-factor factorial experimental designs, illustrating that they are sensitive to the choice of the reference group. We then propose a novel decomposition method to obtain robust decomposition results and use three cases to demonstrate its advantage.

**Results:**

The three decomposition methods differ fundamentally in their allocation of interactions to the contributions of the three factors. In comparison with the existing methods, the new method is robust to the choice of the reference group. Three case studies showed inconsistent attribution results for the two existing methods but robust results for the new method when the choice of the reference population changes.

**Conclusions:**

The proposed method offers robust and more justifiable attribution results compared to the two existing methods. This method could be generalized to attribution of group differences of other health indicators.

## Background

As population aging accelerates worldwide, it is valuable for researchers to quantify the impact of aging on the burden of diseases at global, regional, national and local levels. One important aspect of quantifying the effect of population aging is to attribute death differences between two populations to their differences in population size, age structure, and age-specific mortality rates (ASMR). Such attribution (or decomposition) analysis is key to interpreting death differences across time and place and to developing long-term strategies to improve public health at different levels.

Two approaches have been established and widely used to estimate the contribution of population aging to changes in the total number of population deaths over time [1, 2]. We refer to the approach developed by Bashir et al [1]. as method I and the approach that the Global Burden of Disease (GBD) study group developed by extending the method of Das Gupta for decomposing rate differences as method II [2, 3]. Both approaches ascribe differences in total deaths to the contributions of three factors: (a) age structure of the population (a proxy for population aging), (b) population size, and (c) age-specific mortality rates (ASMR) due to all other reasons (e.g. motorization, urbanization, poverty, environmental pollution, individual behaviors, policy interventions, genetic disorders) [1, 2, 4].

The two approaches provide decompositions with intuitive interpretations in many applications, but some inherent issues with the approaches have not been addressed in the literature. First, as we demonstrate below, the two approaches do not consider how to allocate two-way and three-way interactions between the three factors when decomposing their contributions. Previous studies indicate the presence of an interaction between age structure and ASMR when the mortality rate difference between two populations is attributed to the two factors [5–7]. Second, both methods fail to provide users with useful guidance on the selection of the reference population for a decomposition analysis, but their results heavily depend on such selection. These limitations have not been discussed before despite the fact that both methods are widely used to support health-related decision-making [4, 8, 9].

In this study, we demonstrate that methods I and II generate inconsistent results for the same data when the reference population changes and then propose an alternative decomposition method that overcomes these shortcomings. We apply all three methods to the decomposition of differences in total deaths from unintentional falls (a) between the United States and China in 2017, (b) between the United States in 1990 and the United States in 2017, and (c) between China in 1990 and China in 2017.

## Methods and results

We use formula derivation to elaborate the shortcomings of methods I and II and the development of new method. Suppose we are to decompose the difference in total number of deaths between two populations (*j* = 1, 2). The two populations could be defined by time periods, geographic places, or both. Each population has *p* age groups (*i* = 1, 2, …, *p*). Let *d*_*ij*_, *n*_*ij*_, and *m*_*ij*_ denote the number of deaths, population size, and ASMR for age group *j* of population *i*, respectively. Let *s*_*ij*_ represents the proportion of the *i*^*th*^ age group among the *j*^*th*^ population, (*i* = 1, 2, …, *p*, *j* = 1, 2) (**Table 1**).

**Table 1:** Meaning of mathematical symbols in decomposition formula.

Age group	Group 1 (*j* = 1)				Group 2 (*j* = 2)			
	*d*_i1_	*n*_i1_	*m*_i1_	*s*_i1_	*d*_i2_	*n*_i2_	*m*_i2_	*s*_i2_
1	*d*_11_	*n*_11_	*m*_11_	*s*_11_	*d*_12_	*n*_12_	*m*_12_	*s*_12_
2	*d*_21_	*n*_21_	*m*_21_	*s*_21_	*d*_22_	*n*_22_	*m*_22_	*s*_22_
⁞	⁞	⁞	⁞	⁞	⁞	⁞	⁞	⁞
*p*	*d*_*p*1_	*n*_*p*1_	*m*_*p*1_	*s*_*p*1_	*d*_*p*2_	*n*_*p*2_	*m*_*p*2_	*s*_*p*2_
Total	*D*_1_	*N*_1_	*M*_1_	*S*_1_ = 1	*D*_2_	*N*_2_	*M*_2_	*S*_2_ = 1

Note: *d*_*ij*_, *n*_*ij*_, *m*_*ij*_, and *s*_*ij*_ represent the number of deaths, population size, age-specific mortality rate and proportion of group population among total population for the *ij*^th^ subgroup. *D*_1_ and *D*_2_, *N*_1_ and *N*_2_, *M*_1_ and *M*_2_ represent the total number of deaths, population and crude mortality rate for groups 1 and 2, respectively.

### The two existing decomposition methods

Method I uses six steps to decompose the difference in total deaths between the two populations (*D*_2_—*D*_1_) (note: the first population is selected as the reference for this example) [1]:

- Scale the size of each population to 100 000 persons while retaining the original age structure.
- Based on the scaled population size (100 000 persons), use original ASMRs of each population to calculate the expected total number of deaths for population 1 (*D*_1e_) and population 2 (*D*_2e_), respectively.
- Apply ASMRs from population 1 to the scaled 100 000 persons for population 2 to estimate the expected total number of deaths (*D*ASMR_1_ 2e) for population 2.
- Calculate the relative change (*D*ASMR_1_ 2e-*D*_1e_)/*D*_1e_ and multiply it with *D*_1_ to estimate the contribution of age structure, (*D*ASMR_1_ 2e-*D*_1e_)*D*_1_/*D*_1e_.
- Calculate the relative change (*D*_2e_-*D*ASMR_1_ 2e)/*D*_1e_ and multiply it with *D*_1_ to estimate the contribution of ASMR, (*D*_2e_-*D*ASMR_1_ 2e)*D*_1_/*D*_1e_.
- Subtract the contributions of age structure and ASMR from the difference in deaths between two populations (*D*_2_—*D*_1_) to obtain the contribution of population size, (*D*_2_—*D*_1_)—(*D*ASMR_1_ 2e-*D*_1e_)*D*_1_/*D*_1e_ - (*D*_2e_-*D*ASMR_1_ 2e)*D*_1_/*D*_1e_.

With *AS*_*I*_, *ASMR*_*I*_ and *PS*_*I*_ representing deaths attributed to age structure, ASMR and population size defined by method I, respectively, we derive the following formulas according to the literature (details are provided in the S1 File) [1]:

$${AS}_{I}=\sum_{i=1}^{p}N_{1}(s_{i2}-s_{i1})m_{i1}$$ (1)

$${ASMR}_{I}=\sum_{i=1}^{p}N_{1}s_{i1}(m_{i2}-m_{i1})+\sum_{i=1}^{p}N_{1}(s_{i2}-s_{i1})(m_{i2}-m_{i1})$$ (2)

$${PS}_{I}=\sum_{i=1}^{p}{(N_{2}-N_{1})s}_{i1}m_{i1}+\sum_{i=1}^{p}{(N_{2}-N_{1})s}_{i1}(m_{i2}-m_{i1})+\sum_{i=1}^{p}(N_{2}-N_{1})(s_{i2}-s_{i1})m_{i1}+\sum_{i=1}^{p}\left(N_{2}-N_{1})(s_{i2}-s_{i1})(m_{i2}-m_{i1}\right)$$ (3)

Method II differs slightly from method I by decomposing (*D*_2_—*D*_1_) according to the following five steps (note: the first population is again selected as the reference) [2]:

- Apply the ASMRs and age structure from population 1 to population 2 to calculate the expected total number of deaths, *D*(AS_1_, ASMR_1_) 2e, which denotes the expected deaths of population 2 when it has the same age structure and ASMRs as population 1.
- Apply age-specific mortality rates from population 1 to population 2 to calculate the expected total number of deaths, *D*ASMR_1_ 2e, which represents the expected deaths of population 2 when it has the same ASMRs as population 1.
- (*D*(AS_1_, ASMR_1_) 2e —*D*_1_) reflects the contribution of population size.
- (*D*ASMR_1_ 2e - *D*(AS_1_, ASMR_1_) 2e) denotes the contribution of age structure.
- The contribution of ASMR is thus calculated as (*D*_2_—*D*_1_)—(*D*(AS_1_, ASMR_1_) 2e —*D*_1_)—(*D*ASMR_1_ 2e - *D*(AS_1_, ASMR_1_) 2e).

Using *AS*_*II*_, *ASMR*_*II*_ and *PS*_*II*_ to represent death differences attributed to age structure, ASMR and population size defined by method II, respectively, we have the following formulas according to the literature (note: calculation details are included in the S1 File) [2]:

$${AS}_{II}=\sum_{i=1}^{p}N_{1}(s_{i2}-s_{i1})m_{i1}+\sum_{i=1}^{p}(N_{2}-N_{1})(s_{i2}-s_{i1})m_{i1}$$ (4)

$${ASMR}_{II}=\sum_{i=1}^{p}N_{1}s_{i1}(m_{i2}-m_{i1})+\sum_{i=1}^{p}N_{1}(s_{i2}-s_{i1})(m_{i2}-m_{i1})+\sum_{i=1}^{p}\left(N_{2}-N_{1})s_{i1}(m_{i2}-m_{i1}\right)+\sum_{i=1}^{p}\left(N_{2}-N_{1})(s_{i2}-s_{i1})(m_{i2}-m_{i1}\right)$$ (5)

$${PS}_{II}=\sum_{i=1}^{p}(N_{2}-N_{1})s_{i1}m_{i1}$$ (6)

#### Factorial representation of decomposition methods I and II

The decomposition process can be represented in analogy to the analysis of a three-factor factorial experiment design. We can attribute the difference in total deaths between two populations to the main effect, three two-way interactions and one three-way interaction of population size, age structure, and ASMR. Using population 1 as the reference, the main effects (*M*_*p*_, *M*_*s*_ and *M*_*m*_), the two-way interactions (*I*_*ps*_, *I*_*pm*_, *I*_*sm*_), and the three-way interaction (*I*_*psm*_), where the subscripts *p*, *s* and *m* stand for population size, age structure and ASMR respectively, can be represented as follows:

$$M_{p}=\sum_{i=1}^{p}(N_{2}-N_{1})s_{i1}m_{i1}$$ (7)

$$M_{s}=\sum_{i=1}^{p}N_{1}(s_{i2}-s_{i1})m_{i1}$$ (8)

$$M_{m}=\sum_{i=1}^{p}N_{1}s_{i1}(m_{i2}-m_{i1})$$ (9)

$$I_{ps}=\sum_{i=1}^{p}(N_{2}-N_{1})(s_{i2}-s_{i1})m_{i1}$$ (10)

$$I_{pm}=\sum_{i=1}^{p}\left(N_{2}-N_{1})s_{i1}(m_{i2}-m_{i1}\right)$$ (11)

$$I_{sm}=\sum_{i=1}^{p}N_{1}(s_{i2}-s_{i1})(m_{i2}-m_{i1})$$ (12)

$$I_{psm}=\sum_{i=1}^{p}\left(N_{2}-N_{1})(s_{i2}-s_{i1})(m_{i2}-m_{i1}\right)$$ (13)

Applying the above expressions, the contributions of the three factors defined by methods I and II can be written as:

$${AS}_{I}=M_{s},{ASMR}_{I}=M_{m}+I_{sm},{PS}_{I}=M_{p}+I_{ps}+I_{pm}+I_{psm}$$ (14)

$${AS}_{II}=M_{s}+I_{ps},{ASMR}_{II}=M_{m}+I_{sm}+I_{pm}+I_{psm},{PS}_{II}=M_{p}$$ (15)

Clearly, the allocation schemes of interaction terms to the three factors differ between methods I and II, but are asymmetric for both methods, which leads to the non-robustness of attribution results. A closer look at the terms in (7)-(13) reveals that, when the reference group is switched, all main effects will change both their signs and magnitudes, the two-way interactions will change their magnitudes but not their signs, and the three-way interaction will change their signs but not their magnitudes. For example, the formula for the main effect of population size will change from formula (7) to formula (16) when the reference is altered from population 1 to population 2. As a result of the asymmetric allocation of the interactions, the underlying contribution expressions of population size, age structure and ASMR, as shown in formulas (14) and (15), will change accordingly when the interactions exist and the reference population is changed from population 1 to population 2.

$$M_{p}^{\prime }=\sum_{i=1}^{p}(N_{1}-N_{2})s_{i2}m_{i2}$$ (16)

#### Evaluating the robustness of methods I and II

A simple criterion to evaluate the robustness of methods I and II is to check whether the absolute contributions remain the same but with opposite sign when the reference population changes. Following the principles of the two methods [1, 2], we summarize the formulas for calculating the contributions of the three factors under the two choices of reference populations in **Table 2**. Detailed derivations of these formulas are provided in the S1 File. These formulas indicate that, for both methods, decomposition results vary with the choice of reference population.

**Table 2:** Attributional formulas of three factors for two reference group selection methods, for methods I and II.

Method/ reference group	Formula of decomposition analysis		
	ASMR	Age structure	Population size
Method I			
Group 1	$\sum_{i=1}^{p}N_{1}s_{i2}(m_{i2}-m_{i1})$	$\sum_{i=1}^{p}N_{1}(s_{i2}-s_{i1})m_{i1}$	$\sum_{i=1}^{p}(N_{2}-N_{1})s_{i2}m_{i2}$
Group 2	$\sum_{i=1}^{p}N_{2}s_{i1}(m_{i1}-m_{i2})$	$\sum_{i=1}^{p}N_{2}(s_{i1}-s_{i2})m_{i2}$	$\sum_{i=1}^{p}(N_{1}-N_{2})s_{i1}m_{i1}$
Method II			
Group 1	$\sum_{i=1}^{p}N_{2}s_{i2}(m_{i2}-m_{i1})$	$\sum_{i=1}^{p}N_{2}(s_{i2}-s_{i1})m_{i1}$	$\sum_{i=1}^{p}(N_{2}-N_{1})s_{i1}m_{i1}$
Group 2	$\sum_{i=1}^{p}N_{1}s_{i1}(m_{i1}-m_{i2})$	$\sum_{i=1}^{p}N_{1}(s_{i1}-s_{i2})m_{i2}$	$\sum_{i=1}^{p}(N_{1}-N_{2})s_{i2}m_{i2}$

#### Three illustrative case studies

We present three examples to illustrate how decomposition results depend on the choice of reference population for methods I and II. Specifically, we decomposed the difference in total number of deaths from unintentional falls: (a) between the United States and China in 2017, (b) between 1990 and 2017 in the United States, and (c) between 1990 and 2017 in China. All data were derived from the GBD Study 2017 update (GBD 2017) [10]. Age was divided into ten groups: <5, 5–14, 15–24, 25–34, 35–44, 45–54, 55–64, 65–74, 75–84, and ≥85 years (**Table 3**). Data analysis was performed using R 3.4.0.

**Table 3:** Unintentional fall deaths and population data for China in 1990 and 2017 and the United States in 1990 and 2017 for each age group.

Age group	China in 1990			China in 2017			United States in 1990			United States in 2017		
	Deaths	Mortality	N^#^	Deaths	Mortality	N^#^	Deaths	Mortality	N^#^	Deaths	Mortality	N^#^
< 5	8325	6.47	128597	2383	2.96	80431	113	0.58	19488	57	0.29	19482
5–14	4760	2.30	207415	2026	1.39	145575	56	0.16	36067	38	0.09	41929
15–24	5750	2.22	259216	2355	1.40	168588	296	0.79	37607	235	0.54	43414
25–34	7336	3.69	198541	6492	2.68	242341	508	1.15	44021	402	0.90	44529
35–44	7305	4.60	158775	7385	3.55	208167	611	1.60	38214	572	1.40	40748
45–54	5602	5.63	99503	16473	6.74	244337	746	2.93	25471	1517	3.55	42786
55–64	5635	7.14	78903	14095	8.63	163337	1054	4.97	21222	3000	7.13	42069
65–74	6305	13.64	46230	17849	17.50	101965	1821	10.03	18144	4914	16.75	29346
75–84	8485	49.14	17268	31855	68.54	46477	3720	37.03	10045	9834	68.80	14294
85+	4598	181.62	2532	33860	300.65	11262	4233	135.11	3133	17797	285.11	6242
Total	64102	5.36	1196980	134773	9.54	1412480	13158	5.19	253414	38368	11.81	324839

Source: Global Burden of Disease Collaborative Network. Global Burden of Disease Study 2017 (GBD 2017) Results. Seattle, United States: Institute for Health Metrics and Evaluation (IHME), 2018. http://ghdx.healthdata.org/gbd-results-tool. Accessed November 12, 2018. Note: Mortality means age-specific mortality per 100,000 persons.

^#^: N means the number of population in 1000.

**Fig 1:**
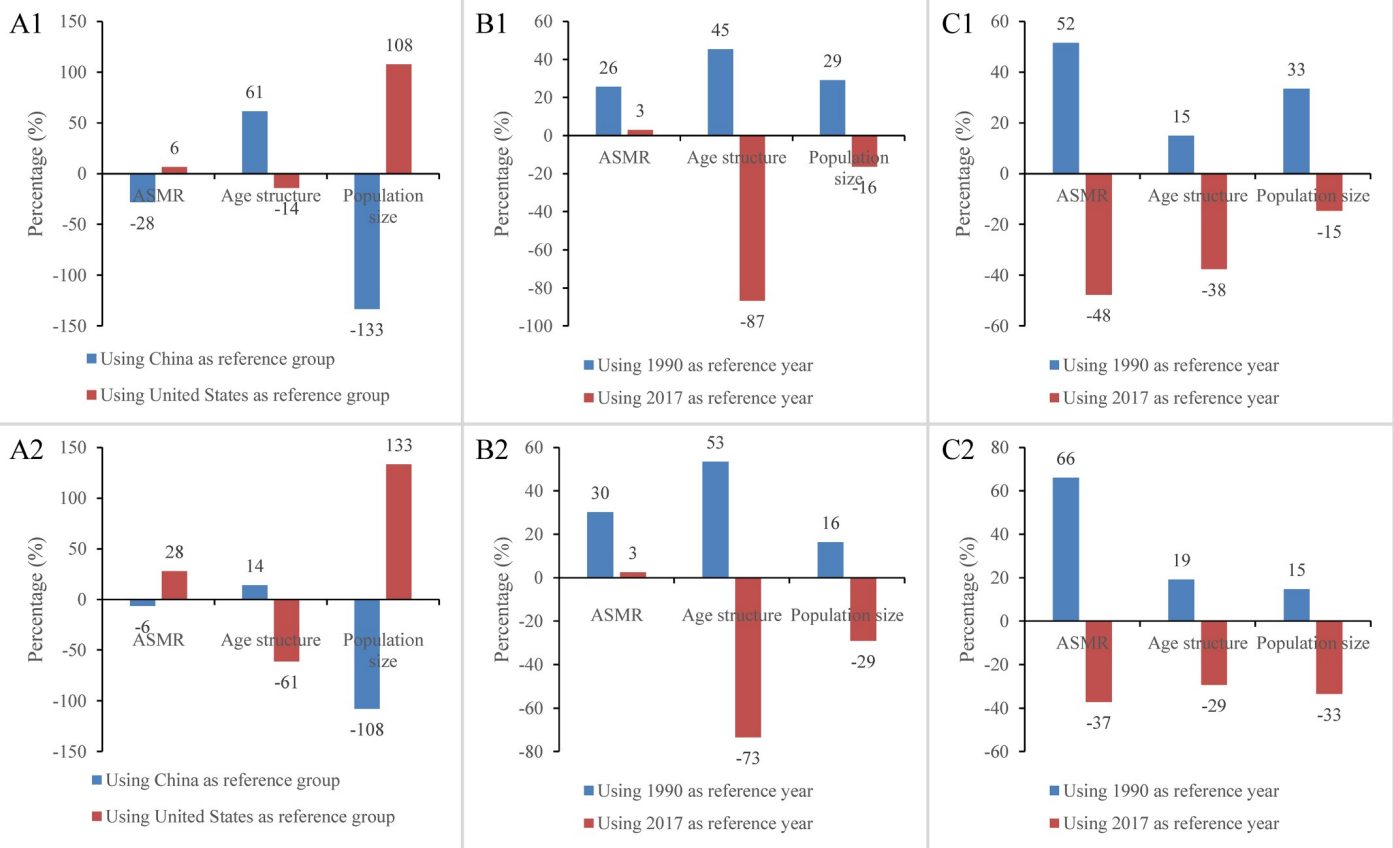
Decomposed contributions of ASMR, age structure and population size to change in total deaths from unintentional falls in the United States and China in 2017, China in 1990 and 2017, and the United States in 1990 and 2017 for different reference population. Notes: Fig 1A1 (Fig 1A2) represents the decomposition results between the United States and China in 2017 from method I (method II); Fig 1B1 (Fig 1B2) represents the decomposition results between China in 1990 and 2017 from method I (method II); Fig 1C1 (Fig 1C2) represents the decomposition results between the United States in 1990 and 2017 from method I (method II).

Case study one. In 2017, approximately 134,773 unintentional fall deaths occurred in China and 38,368 unintentional fall deaths occurred in the United States. The decomposition results of the three factors between China and United States in 2017 changed greatly when the reference population was changed from China to the United States. For method I, the contributions of ASMR, age structure and population size changed from -28% to 6% (deaths: from -27,190 to 6,187), 61% to -14% (deaths: from 59,253 to -13,561), and -133% to 108% (deaths: from -128,467 to 103,778), respectively (note: the signs of contributions reverse when the reference population is switched) (Fig 1A1). We observed changes not only in the signs but also in the magnitudes of contributions. Similarly, the contributions of the three factors changed respectively from -6% to 28% (deaths: from -6,253 to 26,903), 14% to -61% (deaths: from 13,627 to -58,966), and -108% to 133% (deaths: from -103,778 to 128,467) for method II (Fig 1A2).

Case study two. Between 1990 and 2017, the number of unintentional fall deaths rose from 64,102 to 134,773 in China. The estimated contributions of the three factors to the difference between 1990 and 2017 in China changed greatly when we switched the reference year from 1990 to 2017. For method I, the contributions of ASMR, age structure and population size changed respectively from 26% to 3% (deaths: from 18,079 to 2,096), 45% to -87% (deaths: from 32,030 to -61,226), and 29% to -16% (deaths: from 20,562 to -11,541) (Fig 1B1). For Method II, the contributions changed from 30% to 3% (deaths: from 21,333 to 1,776) for ASMR, 53% to -73% (deaths: from 37,796 to -51,885) for age structure, and 16% to -29% (deaths: from 11,541 to -20,562) for population size (Fig 1B2).

Case study three. For the United States, the contributions of the three factors to the differential numbers of unintentional fall deaths between 1990 and 2017 estimated by methods I and II are also sensitive to the choice of the reference year. For method I, the contributions changed from 52% to -48% (deaths: from 13,003 to -12010) for ASMR, 15% to -38% (deaths: from 3,771 to -9,491) for age structure, and 33% to -15% (deaths: from 8,436 to -3,704) for population size (Fig 1C1). For method II, the contributions of the three factors changed respectively from 66% to -37% (deaths: from 16,668 to -9,370), 19% to -29% (deaths: from 4,833 to -7,404), and 15% to -33% (deaths: from 3,709 to -8,436) (Fig 1C2).

### A new decomposition method

When there are no interactions between the three factors, the two established methods generate the same decomposition results. However, in practice, interactions exist in almost all cases. To overcome the deficiencies of methods I and II, we propose a new approach, referred to as method III. This method relies on the principle that a reliable decomposition method should generate stable and consistent results regardless of the choice of reference population. When the reference population is switched, the signs of the contributions of the three factors are expected to reverse, but their magnitudes should remain the same.

Applying this principle, we suggest the three two-way interactions should be equally divided between relevant factors. As there is minimal theoretical guidance about how to allocate the three-way interaction to the three factors, we recommend dividing them equally. The detailed derivations are included in the S1 File. Method III uses four steps to decompose the contributions of the three factors. These steps are illustrated with population 1 as the reference, but results are the same with population 2 as the reference:

- Calculate the main effects of population size (*M*_*p*_), age structure (*M*_*s*_), and ASMR (*M*_*m*_) using formulas (7)–(9).
- Calculate two-way interactions between population size and age structure (*I*_*p*s_), age structure and ASMR (*I*_*sm*_), and population size and ASMR (*I*_*pm*_) using formulas (10)–(12).
- Calculate three-way interaction of the three factors (*I*_*psm*_) using formula (13).
- Using *AS*_*III*_, *ASMR*_*III*_ and *PS*_*III*_ to represent deaths attributed to age structure, population size and ASMR by method III, calculate the contribution of each factor based on the formulas below.

$${AS}_{III}=M_{s}+1/2I_{ps}+1/2I_{sm}+1/3I_{psm}$$ (17)

$${PS}_{III}=M_{p}+1/2I_{ps}+1/2I_{pm}+1/3I_{psm}$$ (18)

$${ASMR}_{III}=M_{m}+1/2I_{pm}+1/2I_{sm}+1/3I_{psm}$$ (19)

The key to the preservation of magnitude and reversed sign is the equal allocation of the two-way interactions. For example, when population 2 is designated as the reference group, (*s*_i2_ –*s*_i1_) in formula (20) is replaced by (*s*_i1_ –*s*_i2_), leading to a reversed sign but a result with the same magnitude. As the three-way interaction also changes its sign but not magnitude when the reference group is switched, the principle holds no matter how we allocate the three-way interaction, i.e., even for unequal allocations. However, equal allocation is a reasonable choice when there is no prior information on the relative importance of the three factors.

$$M_{s}+1/2I_{ps}+1/2I_{sm}=\sum_{i=1}^{p}N_{1}(s_{i2}-s_{i1})m_{i1}+\frac{1}{2}\sum_{i=1}^{p}(N_{2}-N_{1})(s_{i2}-s_{i1})m_{i1}+\frac{1}{2}\sum_{i=1}^{p}N_{1}(s_{i2}-s_{i1})(m_{i2}-m_{i1})=\frac{1}{2}\sum_{i=1}^{p}\left(s_{i2}-s_{i1})(N_{2}m_{i1}+N_{1}m_{i2}\right)$$ (20)

#### Evaluating the performance of method III

Based on the same evaluation criteria mentioned above, we assessed the robustness of method III using the same three case studies. The results suggest that method III generates the same decomposition results regardless of the choice of reference.

**Fig 2:**
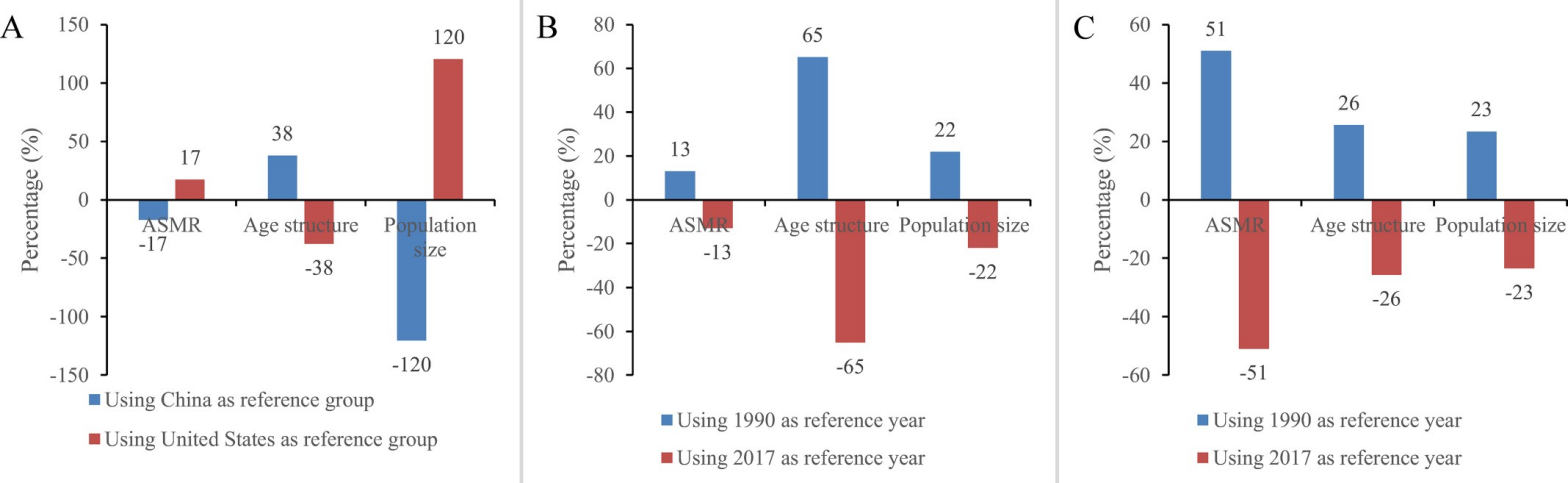
Decomposed contributions of ASMR, age structure and population size to difference in unintentional fall deaths between the United States and China in 2017, China in 1990 and 2017, and the United States in 1990 and 2017 for different reference population for method III. Notes: Fig 2A represents the decomposition results between United States and China in 2017; Fig 2B represents the decomposition results between China in 1990 and 2017; Fig 2C represents the decomposition results between United States in 1990 and 2017.

Case study one. The gap in the total number of unintentional fall deaths between the United States and China in 2017 (-96,404) is explained -17% (-16,615) by ASMR, 38% (36,370) by age structure, and -120% (-116,159) by population size, using US as the reference (Fig 2A) (note: the sum of the percentages of three factors deviates slightly from 100% due to rounded numbers). Using China as the reference, these contribution estimates merely reverse their signs.

Case study two. Using 1990 as the reference, the gap in the total number of unintentional fall deaths between 1990 and 2017 in China (70,671) is explained 13% (9,183) by ASMR, 65% (46,032) by age structure, and 22% (15,456) by population size (Fig 2B). Robust attributions of three factors were observed when the reference was changed.

Case study three. Finally, using 1990 as the reference, the gap in the total number of unintentional fall deaths between 1990 and 2017 in the United States (25,210) is explained 51% (12,848) by ASMR, 26% (6,460) by age structure, and 23% (5,902) by population size (Fig 2C). Similar and robust attributions were observed when the reference was changed.

## Discussion

This study presents evidence toward the two proposed research questions. First, we assessed two existing decomposition methods for quantifying the contributions of ASMR, age structure, and population size to the difference in total number of deaths between two populations. We found that both methods are sensitive to choice of the reference population. By mathematically formulating the decomposition procedures in analogy to a three-factor factorial experiment design, we found that the asymmetric allocations of interactions between relevant factors by both methods are responsible for the inconsistent decomposition results when the reference population is altered. Second, we proposed a new method that overcomes the limitations of the two existing methods.

The three case studies show that the new method III we propose generates different and robust attribution results compared to methods I and II. It overcomes potentially ambiguous or even conflicting interpretations caused by the choice of reference, and thus offers clear and consistent evidence to assist health policy-making.

Our new method (method III) is motivated by previous work by Das Gupta in decomposing a rate difference between two populations into contributions of structural factors (age structure for this study) and structure-specific rate (ASMR in our situation) [3, 5]. However, there are notable differences between Das Gupta’s method and ours. First, our decomposition method applies to differences in the total numbers of events of interest, while Das Gupta’s method was designed to attribute difference in total rates. Second, Das Gupta’s strategy is to average the contributions over all possible decomposition orders of the structural factors (the contribution of structure-specific rates is always decomposed first) and takes the mean as the contribution of the factors. In contrast, our method is built on intuitive and rational allocation of interactions to relevant factors and is a one-step approach that offers more efficient computation. Although modern computers can quickly enumerate all decomposition orders, taking average seems to be an ad hoc way to even out the potential inconsistency among the different decomposition orders. Our new method is theory-driven and totally irrelevant to decomposition order and therefore has robust and ubiquitous interpretation. Last, perhaps because of the complexity of Das Gupta’s method, some previous publications present results based on one decomposition order rather than averaging over all possible decomposition orders [2, 4], leading to conclusions that could be unstable in the presence of interactions. In contrast, our method III is easy to implement and less likely to be misused in practice.

We note that the equal allocation of two-way interactions in our method may not be optimal in aspects other than the robustness of results to the choice of reference population. The equal allocation of the three-way interaction is rather arbitrary, as the allocation proportion does not affect the final attribution. However, given that all two-way interactions are equally allocated to reach robustness, it seems logical to allocate the three-way interaction equally as well. In fact, if Das Gupta’s approach is applied to decomposing rate differences between two groups, we found that its core idea is also to equally divide the interaction of two factors. This idea is similar to the strategy we recommend for attributing differences in total number of deaths between two groups to three factors.

As interactions almost always exist, we recommend use of method III for decomposition analysis, or use of method III in combination with traditional methods. For previous publications that adopted method I or method II to decompose death differences between populations [2, 4, 8, 9], it would be informative to re-analyze those data using method III, or at least to perform sensitivity analyses by changing the reference population to ensure the results are robust.

Although we did not provide uncertainty estimates for the attribution results in our case studies, it is possible to quantify the uncertainty using standard statistical approaches. One possibility is to use a parametric formulation. For example, one could assume the sizes of age groups in each population follow a multinomial distribution with probability parameters *p*_ij_’s, for which *s*_ij_’s are estimates. Given the size of each age group, one could assume the number of death follows either a binomial distribution or a Poisson distribution with the group size as an offset [11, 12]. Direct calculation of variances of the attribution results can be complex due to the presence of products of *s*_ij_ and *m*_ij_; however, parametric bootstrap is a straightforward alternative [13]. In addition, nonparametric bootstrap is another possibility, i.e., sampling individuals from each population with replacement and performing the decomposition analysis repeatedly [13].

We also note that Method III is readily generalizable to attribute differences in a broad spectrum of health outcomes other than number of deaths between two populations to similar factors (structure-specific rates, structural factors, and population size). For example, we could use this method to attribute differences in total incident cases from a specific infectious disease to population size, vaccination status (structural factor), and vaccine-specific incidence rates. In addition, the decomposition method for death rate is essentially the same to that for the number of deaths, the only difference exists in the number of factors to be attributed (two factors for rate difference and three factors for count difference). Therefore, our method is readily generalizable to decomposing difference in mortality rates.

## Conclusion

When interactions between ASMR, age structure, and population size exist, the two existing decomposition methods generate inconsistent results that vary with the choice of reference population. The new method that we propose overcomes this limitation and is a compelling option to attribute a wide range of differences between two populations to contributing factors.

## Supporting information

S1 File The details of formula derivation of three methods. (DOCX)
