# Supplementary material for: A new method to attribute differences in total deaths between groups to population size, age structure and age-specific mortality rate
Source: PLoS One. 2019 May 10;14(5):e0216613. doi: 10.1371/journal.pone.0216613 (PMC6510436; doi:10.1371/journal.pone.0216613)
Supplement: S1 File — (DOCX) [file pone.0216613.s001.docx]

**Online supplement document**

Suppose we are to decompose the difference in total number of deaths between two populations (*j*=1, 2). The two populations could be defined by time periods, geographic places, or both. Each population has *p* age groups (*i* = 1, 2, …, *p*). Let *d_ij_*, *n_ij_*, and *m_ij_* denote the number of deaths, population size, ASMR for the *ij^th^* subgroup, respectively; and *s_ij_* represents the proportion of population size of the *i^th^* group to total population size for the *j^th^* population, respectively, (*i* = 1, 2, …, *p*, *j*=1, 2) (**Table 1**).

**Table 1. Meaning of mathematical symbols in decomposition formula**

| Age group | Population 1 (*j*=1) | | | |  | Population 2 (*j*=2) | | | |
| --- | --- | --- | --- | --- | --- | --- | --- | --- | --- |
|  | *d_i_*_1_ | *n_i_*_1_ | *m_i_*_1_ | *s_i_*_1_ |  | *d_i_*_2_ | *n_i_*_2_ | *m_i_*_2_ | *s_i_*_2_ |
| 1 | *d*_11_ | *n*_11_ | *m*_11_ | *s*_11_ |  | *d*_12_ | *n*_12_ | *m*_12_ | *s*_12_ |
| 2 | *d*_21_ | *n*_21_ | *m*_21_ | *s*_21_ |  | *d*_22_ | *n*_22_ | *m*_22_ | *s*_22_ |
| ⁞ | ⁞ | ⁞ | ⁞ | ⁞ |  | ⁞ | ⁞ | ⁞ | ⁞ |
| *p* | *d_p_*_1_ | *n_p_*_1_ | *m_p_*_1_ | *s_p_*_1_ |  | *d_p_*_2_ | *n_p_*_2_ | *m_p_*_2_ | *s_p_*_2_ |
| Total | *D*_1_ | *N*_1_ | *M*_1_ | *S*_1_=1 |  | *D*_2_ | *N*_2_ | *M*_2_ | *S*_2_=1 |

Note: *d_ij_*, *n_ij_*, *m_ij_*, and *s_ij_* are the number of deaths, population size, age-specific mortality rate and proportion of group population to total population of the *ij*^th^ subgroup. *D*_1_ and *D*_2_, *N*_1_ and *N*_2_, *M*_1_ and *M*_2_ represent total number of deaths, population size and crude mortality rate of populations 1 and 2, respectively.

$D_{1}=\sum_{i=1}^{p} d_{i1}$

$D_{2}=\sum_{i=1}^{p} d_{i2}$

$N_{1}=\sum_{i=1}^{p} n_{i1}$

$N_{2}=\sum_{i=1}^{p} n_{i2}$

$M_{1}=\sum_{i=1}^{p} m_{i1}s_{i1}$

$M_{2}=\sum_{i=1}^{p} m_{i2}s_{i2}$

$m_{ij}=\frac{d_{ij}}{n_{ij}}$

$s_{ij}=\frac{n_{ij}}{N_{j}}$

**1. Formula derivation of method I**

Step 1: calculate the expected deaths in population 1 (*D*_1e_) and population 2 (*D*_2e_) by adjusting the population size of two populations to 100,000 persons:

$D_{1e}=\sum_{i=1}^{p} s_{i1}m_{i1}{10}^{5}$

$D_{2e}=\sum_{i=1}^{p} s_{i2}m_{i2}{10}^{5}$

Step 2: calculate the expected deaths (*D*ASMR_1_ 2e) by applying age-specific mortality rates of population 1 to the simulated population of 100,000 persons for population 2:

$D_{2e}^{{ASMR}_{1}}=\sum_{i=1}^{p} s_{i2}m_{i1}{10}^{5}$

Step 3: calculate the number of deaths attributed to age structure for population 1:

$\left( D_{2e}^{{ASMR}_{1}}-D_{1e} \right)/{D_{1e}\times D_{1}}$

${=\left( \sum_{i=1}^{p} s_{i2}m_{i1}{10}^{5}-\sum_{i=1}^{p} s_{i1}m_{i1}{10}^{5} \right)}/{\sum_{i=1}^{p} s_{i1}m_{i1}{10}^{5}\times\sum_{i=1}^{p} d_{i1}}$

$=\frac{\left( \sum_{i=1}^{p} s_{i2}m_{i1}-\sum_{i=1}^{p} s_{i1}m_{i1} \right)}{\sum_{i=1}^{p} s_{i1}m_{i1}}\times\sum_{i=1}^{p} d_{i1}$

$=\frac{\left( \sum_{i=1}^{p} s_{i2}m_{i1}-\sum_{i=1}^{p} {s_{i1}m}_{i1} \right)}{\sum_{i=1}^{p} \frac{n_{i1}}{N_{1}}\times\frac{d_{i1}}{n_{i1}}}\times\sum_{i=1}^{p} d_{i1}$

$=\frac{\left( \sum_{i=1}^{p} {s_{i2}m}_{i1}-\sum_{i=1}^{p} s_{i1}m_{i1} \right)}{\sum_{i=1}^{p} \frac{d_{i1}}{N_{1}}}\times\sum_{i=1}^{p} d_{i1}$

$=\frac{\left( \sum_{i=1}^{p} {s_{i2}m}_{i1}-\sum_{i=1}^{p} s_{i1}m_{i1} \right)}{\frac{\sum_{i=1}^{p} d_{i1}}{N_{1}}}\times\sum_{i=1}^{p} d_{i1}$

$=\left( \sum_{i=1}^{p} {s_{i2}m}_{i1}-\sum_{i=1}^{p} s_{i1}m_{i1} \right)N_{1}$

$=\sum_{i=1}^{p} N_{1}\left( s_{i2}-s_{i1} \right)m_{i1}$

$=M_{s}$

According to the factorial experiment design of three factors, *M_s_* denotes the main effect of age structure.

Step 4: calculate the number of deaths attributed to ASMR for population 1:

$\left( D_{2e}-D_{2e}^{{ASMR}_{1}} \right)/{D_{1e}\times D_{1}}$

$=\left( \sum_{i=1}^{p} s_{i2}m_{i2}{10}^{5}-\sum_{i=1}^{p} s_{i2}m_{i1}{10}^{5} \right)/{\sum_{i=1}^{p} s_{i1}m_{i1}{10}^{5}\times\sum_{i=1}^{p} d_{i1}}$

$=\frac{\left( \sum_{i=1}^{p} {s_{i2}m}_{i2}-\sum_{i=1}^{p} {s_{i2}m}_{i1} \right)}{\sum_{i=1}^{p} {s_{i1}m}_{i1}}\times\sum_{i=1}^{p} d_{i1}$

$=\frac{\left( \sum_{i=1}^{p} {s_{i2}m}_{i2}-\sum_{i=1}^{p} {s_{i2}m}_{i1} \right)}{\sum_{i=1}^{p} \frac{n_{i1}}{N_{1}}\times\frac{d_{i1}}{n_{i1}}}\times\sum_{i=1}^{p} d_{i1}$

$=\frac{\left( \sum_{i=1}^{p} {s_{i2}m}_{i2}-\sum_{i=1}^{p} s_{i2}m_{i1} \right)}{\sum_{i=1}^{p} \frac{d_{i1}}{N_{1}}}\times\sum_{i=1}^{p} d_{i1}$

$=\frac{\left( \sum_{i=1}^{p} {s_{i2}m}_{i2}-\sum_{i=1}^{p} s_{i2}m_{i1} \right)}{\frac{\sum_{i=1}^{p} d_{i1}}{N_{1}}}\times\sum_{i=1}^{p} d_{i1}$

$=\left( \sum_{i=1}^{p} {s_{i2}m}_{i2}-\sum_{i=1}^{p} s_{i2}m_{i1} \right)N_{1}$

$=\sum_{i=1}^{p} {N_{1}s}_{i2}\left( m_{i2}-m_{i1} \right)$

$=\sum_{i=1}^{p} {N_{1}s}_{i1}\left( m_{i2}-m_{i1} \right)+\sum_{i=1}^{p} N_{1}\left( s_{i2}-s_{i1} \right)\left( m_{i2}-m_{i1} \right)$

$=M_{m}+I_{sm}$

According to the factorial experiment design of the three factors, the first part is the main effect of ASMR (*M_m_*), and the second part is the two-way interaction of ASMR and age structure (*I_sm_*).

Step 5: calculate number of deaths attributed to population size:

$\left( D_{2}-D_{1} \right)-\left( D_{2e}^{{ASMR}_{1}}-D_{1e} \right)/{D_{1e}\times D_{1}}-\left( D_{2e}-D_{2e}^{{ASMR}_{1}} \right)/{D_{1e}\times D_{1}}$

$=\sum_{i=1}^{p} {N_{2}s}_{i2}m_{i2}-\sum_{i=1}^{p} {N_{1}s}_{i1}m_{i1}-\sum_{i=1}^{p} N_{1}\left( s_{i2}-s_{i1} \right)m_{i1}-\sum_{i=1}^{p} {N_{1}s}_{i1}\left( m_{i2}-m_{i1} \right)-\sum_{i=1}^{p} N_{1}\left( s_{i2}-s_{i1} \right)\left( m_{i2}-m_{i1} \right)$

$=\sum_{i=1}^{p} {\left( N_{2}-N_{1} \right)s}_{i1}m_{i1}+\sum_{i=1}^{p} {\left( N_{2}-N_{1} \right)s}_{i1}\left( m_{i2}-m_{i1} \right)+\sum_{i=1}^{p} \left( N_{2}-N_{1} \right)\left( s_{i2}-s_{i1} \right)m_{i1}+\sum_{i=1}^{p} \left( N_{2}-N_{1} \right)\left( s_{i2}-s_{i1} \right)\left( m_{i2}-m_{i1} \right)$

$=S_{p}+I_{ps}+I_{pm}+I_{psm}$

According to the factorial experiment design of the three factors, the first part is the main effect of population size (*M_p_*), and the remaining three parts are two-way interactions of population size and ASMR (*I_pm_*), population size and age structure (*I_ps_*), and three-way interaction of three factors (*I_psm_*), respectively.

**2. Formula derivation of method II**

Step 1: calculate expected deaths ( *D*(AS_1_, ASMR_1_) 2e ) of population 2 by applying both age-specific mortality rates and population structure of population 1 to population 2:

$D_{2e}^{\left( {AS}_{1}, {ASMR}_{1} \right)}=\sum_{i=1}^{p} N_{2}s_{i1}m_{i1}$

Step 2: calculate expected deaths (*D*ASMR_1_ 2e) of population 2 by applying only age-specific mortality rates of population 1 to population 2:

$D_{2e}^{{ASMR}_{1}}=\sum_{i=1}^{p} N_{2}s_{i2}m_{i1}$

Step 3: calculate number of deaths attributed to population size:

$D_{2e}^{\left( {AS}_{1}, {ASMR}_{1} \right)}-D_{1}$

$=\sum_{i=1}^{p} N_{2}s_{i1}m_{i1}-\sum_{i=1}^{p} N_{1}s_{i1}m_{i1}$

$=\sum_{i=1}^{p} \left( N_{2}-N_{1} \right){s_{i1}m}_{i1}$

$=M_{p}$

According to the factorial experiment design of the three factors, *M_p_* represents the main effect of population size.

Step 4: calculate number of deaths attributed to age structure:

$\left( D_{2e}^{{ASMR}_{1}}-D_{1} \right)-\left( D_{2e}^{\left( {AS}_{1}, {ASMR}_{1} \right)}-D_{1} \right)$

$=\left( D_{2e}^{{ASMR}_{1}}-D_{2e}^{\left( {AS}_{1}, {ASMR}_{1} \right)} \right)$

$=\sum_{i=1}^{p} N_{2}s_{i2}m_{i1}-\sum_{i=1}^{p} N_{2}s_{i1}m_{i1}$

$=\sum_{i=1}^{p} N_{2}\left( s_{i2}-s_{i1} \right)m_{i1}$

$=\sum_{i=1}^{p} N_{1}\left( s_{i2}-s_{i1} \right)m_{i1}+\sum_{i=1}^{p} \left( N_{2}-N_{1} \right)\left( s_{i2}-s_{i1} \right)m_{i1}$

$=M_{s}+I_{ps}$

According to the factorial experiment design of the three factors, *M_s_* denotes the main effect of age structure and *I_ps_* represents the two-way interaction of population size and age structure.

Step 5: calculate number of deaths attributed to ASMR:

$\left( D_{2}-D_{1} \right)-\left( D_{2e}^{{ASMR}_{1}}-D_{2e}^{\left( {AS}_{1}, {ASMR}_{1} \right)} \right)-\left( D_{2e}^{\left( {AS}_{1}, {ASMR}_{1} \right)}-D_{1} \right)$

$=\left( D_{2}-D_{2e}^{{ASMR}_{1}} \right)$

$=\sum_{i=1}^{p} N_{2}s_{i2}m_{i2}-\sum_{i=1}^{p} N_{2}s_{i2}m_{i1}$

$=\sum_{i=1}^{p} {N_{2}s}_{i2}\left( m_{i2}-m_{i1} \right)$

$=\sum_{i=1}^{p} N_{1}s_{i1}(m_{i2}-m_{i1})+\sum_{i=1}^{p} N_{1}\left( s_{i2}-s_{i1} \right)\left( m_{i2}-m_{i1} \right)+\sum_{i=1}^{p} \left( N_{2}-N_{1} \right)s_{i1}\left( m_{i2}-m_{i1} \right)+\sum_{i=1}^{p} \left( N_{2}-N_{1} \right)\left( s_{i2}-s_{i1} \right)\left( m_{i2}-m_{i1} \right)$

$={{S_{m}+I}_{sm}+I}_{pm}+I_{psm}$

According to the factorial experiment design of the three factors, *S_m_* is the simple effect of ASMR, and the remaining three parts are two-way interactions of ASMR and age structure (*I_sm_*), population size and ASMR (*I_pm_*), and the three-way interaction of the three factors (*I_psm_*), respectively.

**3. Assessing the performance of methods I and II**

**3.1. Consistency between methods I and II**

Using *AS_I_* (*AS_II_*), *ASMR_I_* (*ASMR_II_*) and *PS_I_* (*PS_II_*) to represent the number of deaths attributed to age structure, ASMR and population size defined by method I (II), we calculate differences in the contribution of the three factors between the two methods:

$${AS}_{I}-{AS}_{II}=M_{s}-\left( M_{s}+I_{ps} \right)=-I_{ps}$$

$${ASMR}_{I}-{ASMR}_{II}=M_{m}+I_{sm}-\left( M_{m}+I_{sm}+I_{pm}+I_{psm} \right)=-I_{pm}-I_{psm}$$

$${PS}_{I}-{PS}_{II}=M_{p}+I_{ps}+I_{pm}+I_{psm}-M_{p}=I_{ps}+I_{pm}+I_{psm}$$

The results show that the decomposition results from methods I and II are different unless there are no interactions between two and three factors, a situation that would be extremely rare in practice.

**3.2. Stability of method I**

Using *AS' I*, *ASMR' I* and *PS' I* to represent the number of deaths attributed to age structure, ASMR and population size when selecting population 2 as the reference, , respectively, we calculate as follows:

${{AS}_{I}^{'}=\left( D_{1e}^{{ASMR}_{2}}-D_{2e} \right)}/{D_{2e}\times D_{2}}$

$=\left( \sum_{i=1}^{p} s_{i1}m_{i2}{10}^{5}-\sum_{i=1}^{p} s_{i2}m_{i2}{10}^{5} \right)/{\sum_{i=1}^{p} s_{i2}m_{i2}{10}^{5}\times\sum_{i=1}^{p} d_{i2}}$

$=\frac{\left( \sum_{i=1}^{p} s_{i1}m_{i2}-\sum_{i=1}^{p} {s_{i2}m}_{i2} \right)}{\sum_{i=1}^{p} \frac{n_{i2}}{N_{2}}\times\frac{d_{i2}}{n_{i2}}}\times\sum_{i=1}^{p} d_{i2}$

$=\left( \sum_{i=1}^{p} {s_{i1}m}_{i2}-\sum_{i=1}^{p} s_{i2}m_{i2} \right)N_{2}$

$=\sum_{i=1}^{p} N_{2}\left( s_{i1}-s_{i2} \right)m_{i2}$

${{ASMR}_{I}^{'}=\left( D_{1e}-D_{1e}^{{ASMR}_{2}} \right)}/{D_{2e}\times D_{2}}$

$=\left( \sum_{i=1}^{p} s_{i1}m_{i1}{10}^{5}-\sum_{i=1}^{p} s_{i1}m_{i2}{10}^{5} \right)/{\sum_{i=1}^{p} s_{i2}m_{i2}{10}^{5}\times\sum_{i=1}^{p} d_{i2}}$

$=\frac{\left( \sum_{i=1}^{p} s_{i1}m_{i1}-\sum_{i=1}^{p} {s_{i1}m}_{i2} \right)}{\sum_{i=1}^{p} \frac{n_{i2}}{N_{2}}\times\frac{d_{i2}}{n_{i2}}}\times\sum_{i=1}^{p} d_{i2}$

$=\left( \sum_{i=1}^{p} {s_{i1}m}_{i1}-\sum_{i=1}^{p} s_{i1}m_{i2} \right)N_{2}$

$=\sum_{i=1}^{p} {N_{2}s}_{i1}\left( m_{i1}-m_{i2} \right)$

${PS}_{I}^{'}=\left( D_{1}-D_{2} \right)-\left( D_{1e}^{{ASMR}_{2}}-D_{2e} \right)/{D_{2e}\times D_{2}}-\left( D_{1e}-D_{1e}^{{ASMR}_{2}} \right)/{D_{2e}\times D_{2}}$

$=\sum_{i=1}^{p} {N_{1}s}_{i1}m_{i1}-\sum_{i=1}^{p} {N_{2}s}_{i2}m_{i2}-\sum_{i=1}^{p} N_{2}\left( s_{i1}-s_{i2} \right)m_{i2}- \sum_{i=1}^{p} {N_{2}s}_{i1}\left( m_{i1}-m_{i2} \right)$

$=\sum_{i=1}^{p} {{\left( N_{1}-N_{2} \right)s}_{i1}m}_{i1}$

Calculating differences in decomposition results of the three factors by changing the reference population from population 1 to 2, we derive the following formulas:

${AS}_{I}^{'}-{AS}_{I}=\sum_{i=1}^{p} \left( s_{i1}-s_{i2} \right)(N_{2}m_{i2}-N_{1}m_{i1})$

${ASMR}_{I}^{'}-{ASMR}_{I}=\sum_{i=1}^{p} \left( m_{i1}-m_{i2} \right)({N_{2}s}_{i1}-{N_{1}s}_{i2})$

${PS}_{I}^{'}-{PS}_{I}=\sum_{i=1}^{p} \left( N_{1}-N_{2} \right)(s_{i1}m_{i1}-s_{i2}m_{i2})$

The decomposition results are different after changing the reference population unless there are no interaction between three factors, a situation that is extremely rare in practice.

**3.3. Stability of method II**

Using *AS' II*, *ASMR' II* and *PS' II* to represent the number of deaths attributed to age structure, ASMR and population size when selecting population 2 as the reference, respectively, we calculate as follows:

${PS}_{II}^{'}=D_{1e}^{\left( {AS}_{2}, {ASMR}_{2} \right)}-D_{2}$

$=\sum_{i=1}^{p} N_{1}s_{i2}m_{i2}-\sum_{i=1}^{p} N_{2}s_{i2}m_{i2}$

$=\sum_{i=1}^{p} \left( N_{1}-N_{2} \right){s_{i2}m}_{i2}$

${AS}_{II}^{'}=\left( D_{1e}^{{ASMR}_{2}}-D_{2} \right)-\left( D_{1e}^{\left( {AS}_{2}, {ASMR}_{2} \right)}-D_{2} \right)$

$=\left( D_{1e}^{{ASMR}_{2}}-D_{1e}^{\left( {AS}_{2}, {ASMR}_{2} \right)} \right)$

$=\sum_{i=1}^{p} N_{1}s_{i1}m_{i2}-\sum_{i=1}^{p} N_{1}s_{i2}m_{i2}$

$=\sum_{i=1}^{p} N_{1}\left( s_{i1}-s_{i2} \right)m_{i2}$

${ASMR}_{II}^{'}=\left( D_{1}-D_{2} \right)-\left( D_{1e}^{{ASMR}_{2}}-D_{1e}^{\left( {AS}_{2}, {ASMR}_{2} \right)} \right)-\left( D_{1e}^{\left( {AS}_{2}, {ASMR}_{2} \right)}-D_{2} \right)$

$=\left( D_{1}-D_{1e}^{{ASMR}_{2}} \right)$

$=\sum_{i=1}^{p} N_{1}s_{i1}m_{i1}-\sum_{i=1}^{p} N_{1}s_{i1}m_{i2}$

$=\sum_{i=1}^{p} {N_{1}s}_{i1}\left( m_{i1}-m_{i2} \right)$

Compared with the original results of selecting population 1 as the reference, we obtain:

${AS}_{II}^{'}-{AS}_{II}=\sum_{i=1}^{p} \left( s_{i1}-s_{i2} \right)(N_{1}m_{i2}-N_{2}m_{i1})$

${ASMR}_{II}^{'}-{ASMR}_{II}=\sum_{i=1}^{p} \left( m_{i1}-m_{i2} \right)({N_{1}s}_{i1}-{N_{2}s}_{i2})$

${PS}_{II}^{'}-{PS}_{II}=\sum_{i=1}^{p} \left( N_{1}-N_{2} \right)(s_{i2}m_{i2}-s_{i1}m_{i1})$

The decomposition results are different after changing the reference population unless there are no interactions between the three factors.

**4. Formula derivation of method III**

To overcome the deficiencies of methods I and II, we developed an innovative approach, method III, based on the principle that a reliable decomposition method should generate stable and consistent results regardless of the change of reference population.

Based on the factorial experiment design of three factors and methods I and II, we first calculate the main effect, two-way interactions, and three-way interactions between population size, age structure and ASMR on the difference in total deaths between the two populations. We use population 1 as the reference first, and then we conduct decomposition analysis using population 2 as the reference.

Using population 1 as the reference, main effects (*M_p_*, *M_s_* and *M_m_*) and two-way and three-way interactions (*I_ps_*, *I_pm_*, *I_sm_* and *I_psm_*) of the three factors are calculated as follows:

$M_{p}=\sum_{i=1}^{p} {{\left( N_{2}-N_{1} \right)s}_{i1}m}_{i1}$

$M_{s}=\sum_{i=1}^{p} N_{1}\left( s_{i2}-s_{i1} \right)m_{i1}$

$M_{m}=\sum_{i=1}^{p} {N_{1}s}_{i1}\left( m_{i2}-m_{i1} \right)$

$I_{ps}=\sum_{i=1}^{p} \left( N_{2}-N_{1} \right)\left( s_{i2}-s_{i1} \right)m_{i1}$

$I_{pm}=\sum_{i=1}^{p} \left( N_{2}-N_{1} \right)s_{i1}\left( m_{i2}-m_{i1} \right)$

$I_{sm}=\sum_{i=1}^{p} N_{1}\left( s_{i2}-s_{i1} \right)\left( m_{i2}-m_{i1} \right)$

$I_{psm}=\sum_{i=1}^{p} \left( N_{2}-N_{1} \right)\left( s_{i2}-s_{i1} \right)\left( m_{i2}-m_{i1} \right)$

Using population 2 as the reference, the formulas are calculated as follows:

$M_{p}^{'}=\sum_{i=1}^{p} {{\left( N_{1}-N_{2} \right)s}_{i2}m}_{i2}$

$M_{s}^{'}=\sum_{i=1}^{p} N_{2}\left( s_{i1}-s_{i2} \right)m_{i2}$

$M_{m}^{'}=\sum_{i=1}^{p} {N_{2}s}_{i2}\left( m_{i1}-m_{i2} \right)$

$I_{ps}^{'}=\sum_{i=1}^{p} \left( N_{1}-N_{2} \right)\left( s_{i1}-s_{i2} \right)m_{i2}$

$I_{pm}^{'}=\sum_{i=1}^{p} \left( N_{1}-N_{2} \right)s_{i2}\left( m_{i1}-m_{i2} \right)$

$I_{sm}^{'}=\sum_{i=1}^{p} N_{2}\left( s_{i1}-s_{i2} \right)\left( m_{i1}-m_{i2} \right)$

$I_{psm}^{'}=\sum_{i=1}^{p} \left( N_{1}-N_{2} \right)\left( s_{i1}-s_{i2} \right)\left( m_{i1}-m_{i2} \right)$

The contribution of each factor should include its main effect and partly of related interactions.

(1) Suppose *a*%, *b*% and *c*% of the two-way interaction between population size and age structure, population size and ASMR, and age structure and ASMR are allocated to the first factor, respectively. Accordingly, (100-*a*)%, (100-*b*)% and (100-*c*)% of the three two-way interactions are allocated to the second factor.

And (2) suppose *d*_1_%, *d*_2_% and (100- *d*_1_-*d*_2_)% of the three-way interaction are allocated to population size, age structure, and ASMR, respectively.

Using *AS_III_* (*AS' III*), *ASMR_III_* (*ASMR' III*) and *PS_III_* (*PS' III*) to represent the number of deaths attributed to age structure, ASMR and population size defined by method III when using population 1 (2) as reference, the contributions of the three factors can be calculated as follows:

$${PS}_{III}=M_{p}+a\%I_{ps}+b\%I_{pm}+d_{1}\%I_{psm}$$

$${{AS}_{III}=M}_{s}+\left( 100-a \right)\%I_{ps}+c\%I_{sm}+d_{2}\%I_{psm}$$

$${ASMR}_{III}=M_{m}+\left( 100-b \right)\%I_{pm}+\left( 100-c \right)\%I_{sm}+\left( 100-d_{1}-d_{2} \right)\%I_{psm}$$

$${{PS}_{III}^{'}=M}_{p}^{'}+a\%I_{ps}^{'}+b\%I_{pm}^{'}+d_{1}\%I_{psm}^{'}$$

$${{AS}_{III}^{'}=M}_{s}^{'}+\left( 100-a \right)\%I_{ps}^{'}+c\%I_{sm}^{'}+d_{2}\%I_{psm}^{'}$$

$${{ASMR}_{III}^{'}=M}_{m}^{'}+\left( 100-b \right)\%I_{pm}^{'}+(100-c)\%I_{sm}^{'}+\left( 100-d_{1}-d_{2} \right)\%I_{psm}^{'}$$

According to the principle above, the decomposition results should remain unchanged in absolute value when the reference population changes, so we have a group of three equations:

$\left\{ \begin{aligned} {PS}_{III}\equiv-{PS}_{III}^{'} \\ {AS}_{III}\equiv-{AS}_{III}^{'} \\ {ASMR}_{III}\equiv-{ASMR}_{III}^{'} \end{aligned} \right.$

Through formula derivation, we have three simplified equations:

$\left\{ \begin{aligned} \sum_{i=1}^{p} \left( N_{2}-N_{1} \right)\left[ \left( s_{i1}m_{i1}-s_{i2}m_{i2} \right)\left( 100-a-b \right)\%+\left( s_{i2}m_{i1}-s_{i1}m_{i2} \right)\left( a-b \right)\% \right]\equiv0 \\ \sum_{i=1}^{p} \left( s_{i2}-s_{i1} \right)\left[ \left( {N_{2}m_{i1}-N}_{1}m_{i2} \right)\left( 100-a-c \right)\%+\left( N_{1}m_{i1}-N_{2}m_{i2} \right)\left( a-c \right)\% \right]\equiv0 \\ \sum_{i=1}^{p} \left( m_{i2}-m_{i1} \right)\left[ \left( {{N_{2}s_{i2}-N}_{1}s}_{i1} \right)\left( 100-b-c \right)\%+\left( N_{1}s_{i2}-N_{2}s_{i1} \right)\left( b-c \right)\% \right]\equiv0 \end{aligned} \right.$

These three equations cannot be true all the time unless *a*, *b*, and *c* all equal 50.

The three equations have no requirements for *d*_1_ and *d*_2_. Given there is no theoretical guidance to allocate the three-way interaction of three factors, we divide it equally, *d*_1_=*d*_2_=1/3×100.

Last, we have the attribution formulas of the three factors as follows when using population 1 as reference:

$${{PS}_{III}=M}_{p}+1/2I_{pm}+1/2I_{ps}+1/3I_{psm}$$

$${{AS}_{III}=M}_{s}+1/2I_{sm}+1/2I_{ps}+1/3I_{psm}$$

$${{ASMR}_{III}=M}_{m}+1/2I_{pm}+1/2I_{sm}+1/3I_{psm}$$
